# Serum hCG Levels following the Ovulatory Injection: Associations with Patient Weight and Implantation Time

**DOI:** 10.1155/2015/520714

**Published:** 2015-10-26

**Authors:** Dorette J. Noorhasan, Peter G. McGovern, Michael Cho, Aimee Seungdamrong, Khaliq Ahmad, David H. McCulloh

**Affiliations:** ^1^Division of Reproductive Endocrinology and Infertility, Department of Obstetrics, Gynecology and Women's Health, New Jersey Medical School, UMDNJ, Newark, NJ 07103, USA; ^2^University Reproductive Associates, Hasbrouck Heights, NJ 07604, USA; ^3^Fertility Specialists of Texas, 5757 Warren Parkway, Suite No. 300, Frisco, TX 75034, USA; ^4^Department of Obstetrics and Gynecology, Saint Luke's Roosevelt Hospital, New York, NY 10019, USA; ^5^Department of Obstetrics and Gynecology, Texas Tech University Health Sciences Center School of Medicine, Lubbock, TX 79430, USA; ^6^NYU Fertility Center, New York University Langone Medical Center, New York, NY 10016, USA

## Abstract

*Objective*. To test if serum hCG levels the morning after the ovulatory hCG injection correlate with (1) retrieval efficiency, (2) oocyte maturity, (3) embryo quality, (4) pregnancy, and/or (5) time to implantation in patients undergoing in vitro fertilization (IVF) with intracytoplasmic sperm injection (ICSI). *Design*. Retrospective cohort analysis. *Setting*. University-based IVF clinic. *Patient(s)*. All IVF/ICSI cycles from April 2005 to February 2008 whose hCG administration was confirmed (*n* = 472 patients). *Intervention(s)*. Serum hCG was measured the morning following the ovulatory injection, on the 16th day following retrieval, and repeated on day 18 for those with positive results. *Main Outcome Measure(s)*. Number of follicles on the day of hCG injection, number of oocytes retrieved, maturity of oocytes, embryo quality, pregnancy outcome, and time to implantation. *Result(s)*. hCG levels did not correlate with retrieval efficiency, oocyte maturity, embryo quality, or pregnancy. Postinjection hCG levels were inversely associated with patient weight and time to implantation. *Conclusion(s)*. No correlation was found between hCG level and any parameter of embryo quality. Patient weight affected hCG levels following hCG injection and during the early period of pregnancy following implantation. No association between postinjection hCG level and time of implantation (adjusted for patient weight) was apparent.

## 1. Introduction

Traditionally 10,000 IU of hCG is administered to cause final oocyte maturation and ovulation in patients undergoing in vitro fertilization (IVF). It is well established that hCG can mimic the midcycle LH surge [1]. It is clear that the ovulatory injection of hCG is required to assure that oocytes will be retrieved at time of retrieval [2–5], thus avoiding “empty follicle syndrome.” In our practice, we have patients returning the morning after administering 10,000 IU of hCG so that we may assess their serum hCG level and to help determine if the patients correctly administered the injection. Although it is clear that some level of serum hCG after the ovulatory injection is necessary for oocyte retrieval, it is not clear whether a specific threshold level of serum hCG is required to permit successful IVF outcome. In addition, we chose to examine if the patient's weight (a correlate of the patient's volume of distribution) for this fixed dose of administration and for any endogenous hCG production affects circulating hCG levels and outcomes.

The objectives of this study were to evaluate if the serum hCG levels in blood drawn the morning following ovulatory injection of hCG correlate with (1) retrieval efficiency, (2) maturity of the retrieved oocytes, (3) embryo developmental extent and quality, (4) incidence of pregnancy, and/or (5) time to implantation in patients undergoing in vitro fertilization (IVF) with intracytoplasmic sperm injection (ICSI).

## 2. Materials and Methods

### 2.1. Study Population

The Rutgers-New Jersey Medical School (formerly UMDNJ) Institutional Review Board approved this study (IRB # 0120070090). A retrospective chart review of all patients undergoing IVF with ICSI between April 2005 and February 2008 at a university-affiliated reproductive endocrinology clinic was conducted. Follicular growth was monitored by ultrasound and serum estradiol measurements. Follicular growth was considered sufficient when ultrasound monitoring revealed at least 2 follicles with a mean diameter of 16 mm or larger. Each patient was instructed to administer an intramuscular injection of 10,000 IU of hCG later that night for final oocyte maturation and ovulatory stimulation. Oocyte retrieval was scheduled for 34 hours after the injection. All patients were instructed to return for a blood draw between 7:00 and 8:00 am on the morning following the injection, to confirm that the hCG medication had been properly administered (6 ± 2 hours later). Serum hCG was assessed by chemiluminescent assay (Immulite 1000, Siemens, Deerfield, IL). A total of 472 IVF cycles in 367 patients were found to have confirmatory values of serum hCG determined the morning after the hCG injection. Of these 367 patients, 280 underwent only one retrieval, 72 patients underwent 2 cycles, 12 patients underwent 3 retrievals, and 3 patients underwent 4 retrievals. Records were reviewed to determine the number of follicles (~10 mm and larger) seen on ultrasound monitoring the morning prior to hCG injection, the oocytes that did not contain a germinal vesicle (GV) (i.e., mature oocytes), the developmental extent and quality of embryos that were transferred, and the incidence of pregnancy.

### 2.2. Retrieval Efficiency

The number of oocytes retrieved was divided by the number of follicles observed in the ovaries on the final day of ultrasound monitoring (the morning prior to injection of hCG). This ratio served to estimate the efficiency of retrieving oocytes from the follicles (the proportion of follicles yielding oocytes).

### 2.3. Oocyte Maturity

The number of oocytes with a germinal vesicle at the time of oocyte clean-up prior to intracytoplasmic sperm injection (ICSI) was determined. The percentage of oocytes that did* not* contain a germinal vesicle was used as an estimate of the number of mature oocytes (that had begun the maturation), since this was the only criterion that we used to determine the oocytes on which we performed ICSI.

### 2.4. Embryo Developmental Extent and Quality

**Figure 1:**
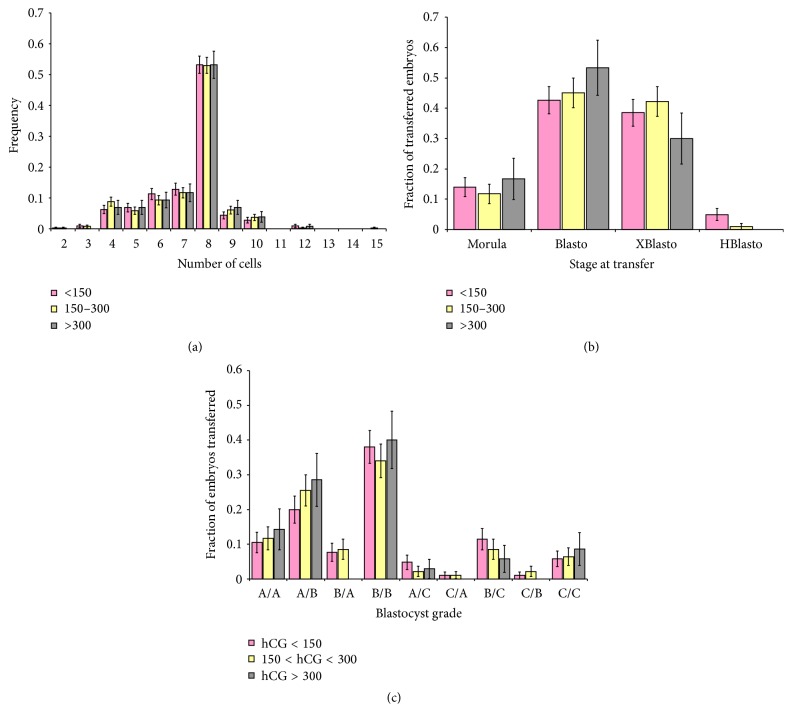
Postovulatory serum hCG levels and embryo quality. Bars indicate the incidence (frequency) of embryos with specified numbers of cells transferred on day 3 (a), embryo stage transferred on day 5 (b), and embryo grade transferred on day 5 (c). Error bars represent standard error. Postovulatory serum hCG levels were not associated with the number of cells in day 3 embryos (Contingency Chi Squared, *p* = 0.972) (a), the embryo stage in day 5 transfers (Contingency Chi Squared, *p* = 0.399) (b), nor the blastocyst grade in day 5 transfers (Contingency Chi Squared, *p* = 0.933).

The developmental extent as well as embryo quality was assessed for all embryos transferred back to the patient's uterus. For embryos transferred on day 3, the number of cells was counted and served as an indicator of the extent of embryo development. Grades for embryos transferred on day 3 (A, B, and C) were based on a combination of cell sizes (appropriate equality of divisions) and on fragmentation (less fragmentation leading to higher scores). For embryos transferred on day 5, the extent of blastocyst development was determined: morulae (M) with no sign of blastocele formation, blastocysts (B) with a blastocele that had not begun to expand, blastocysts that had expanded (XB), and blastocysts undergoing hatching (H). In addition, scores indicative of blastocyst quality were evaluated using a two-letter grade indicative of the number of cells in the inner cell mass (A > 7, B 4–7, and C < 4) and the number of cells seen in one focal plane at the equator of the blastocyst (A > 8, B 4–8, and C < 4). The first letter presented in Figure 1(c) denotes the number of cells in the inner cell mass.

### 2.5. Incidence of Clinical Pregnancy

Serum hCG levels were determined on the 16th day following oocyte retrieval to determine if pregnancy had occurred. If the value on day 16 following oocyte retrieval was greater than 5.3 mIU/mL (the threshold of the assay for detecting pregnancy), serum hCG level was repeated on the 18th day following oocyte retrieval to look for a rise in serum hCG values 2 days after the initial measurement. An ultrasound examination was performed 4 weeks following the oocyte retrieval if hCG levels continued to rise after days 16 and 18. Clinical pregnancy was defined as the presence of at least one fetal sac in the uterus detected using ultrasound.

### 2.6. Time to Implantation

Only those patients with hCG levels that increased between day 16 and day 18 were evaluated for time of implantation (*n* = 113). The time of implantation was determined by extrapolation of the linear regression line relating the two ln [hCG]'s (for day 16 and 18 serum hCG values) versus time to the value at which [hCG] equaled 10 mIU/mL for each fetal sac seen, similar to a method reported previously [6]. A serum hCG value of 10 mIU/mL was arbitrarily chosen for each fetal sac because, in previous examinations, this choice of threshold yielded a relative minimum in the standard deviation of estimated implantation times (it is also about twice the manufacturer's lower limit of detecting a pregnancy (5.2 IU/L)).

### 2.7. Statistical Analysis

Linear regression analyses were performed to evaluate the correlation between serum hCG and the number of oocytes retrieved per follicle scanned, maturity of the oocytes, clinical pregnancy rate, and time to implantation. Contingency Chi Squared tests were performed to evaluate the association between serum hCG and embryo quality. A *p* value of <0.05 was considered to be statistically significant.

## 3. Results

Serum hCG levels determined the morning following the hCG injection averaged 202 ± 122 IU/L. Values of serum hCG varied widely ranging from 35 to 623 IU/L.

### 3.1. Efficiency of Oocyte Retrieval

The percentage of oocytes retrieved per follicle scanned was 89 ± 29% (Figure 1). Values for efficiency of oocyte retrieval varied widely from 27% to 260%. (Values exceeding 100% probably occurred due to difficulty with ultrasound visualization of the ovaries.) Values for efficiency of oocyte retrieval were not significantly associated with the serum hCG level determined the morning following the hCG injection (*R* = 0.073; *p* = 0.37).

### 3.2. Oocyte Maturity

The percentage of mature oocytes was 88 ± 13.5%. The percentage of oocytes that were mature varied widely and ranged from 43% to 100%. Values for the percentage of oocytes that were mature were not significantly associated with the serum hCG levels determined the morning following the hCG injection (*R* = 0.11; *p* = 0.18).

### 3.3. Embryo Developmental Extent and Quality

Of the 472 patients, 343 patients underwent embryo transfer (total of 822 embryos) on day 3 and 129 underwent embryo transfer (total of 234 embryos) on day 5. Based upon the distribution of hCG values, we divided the patients undergoing day 3 transfer into three categories representing three groups defined as low, moderate, and high levels of hCG (<150 mIU/mL with 134 patients and 318 embryos, 150–300 mIU/mL with 156 patients and 376 embryos, and >300 mIU/mL with 53 patients and 128 embryos). The number of cells in each embryo was assessed for embryos transferred on day 3. The distribution of cell numbers, indicative of developmental extent, is shown in Figure 1(a). Embryos with 8 cells were most common in all 3 categories with other cell numbers less represented in all categories (Figure 1(a)). The similarity of the three hCG categories suggests that there was no significant difference in the developmental extent in the three groups. The distributions were not significantly different when compared using Contingency Chi Squared: *χ* ^2^ = 4.5 with 12 degrees of freedom; *p* = 0.972. This indicates that there was no association between the hCG groups and the distribution of numbers of cells. Hence, in day 3 embryos, the developmental extent was not associated with the serum hCG level determined the morning following the hCG injection (Figure 1(a)).

Embryo grades, reflecting evenness of cell divisions, and lack of fragmentation were compared for the same three groups of patients examined for cell numbers. The distributions of grades were not significant (Contingency Chi Squared: *χ* ^2^ = 4.5 with 3 degrees of freedom; *p* = 0.2).

Similarly, the extent of blastocyst development was assessed for embryos transferred on day 5. Blastocysts that had no expanded blastocele (Blasto) and blastocysts that had expanded blastocele (XBlasto) were predominant in all three categories. Comparison of the distributions of blastocyst development yielded no significant differences (Contingency Chi Squared: *χ* ^2^ = 6.22 with 6 degrees of freedom; *p* = 0.399). Therefore, the stages of the blastocysts transferred on day 5 were not associated with the hCG groups. Hence, blastocyst stages were not associated with serum hCG levels determined the morning following the hCG injection (Figure 1(b)).

The quality of blastocysts was assessed for embryos transferred on day 5. Based upon the distribution of hCG values, we divided the 129 patients undergoing day 5 transfer into three categories representing three groups defined as low, moderate, and high levels of hCG (<150 mIU/mL with 59 patients with 105 embryos, 150–300 mIU/mL with 52 patients with 94 embryos, and >300 mIU/mL with 18 patients with 35 embryos). Blastocysts graded A/B and B/B were predominant in all three hCG categories. Comparison of the distributions of blastocyst grades yielded no significant differences (Contingency Chi Squared: *χ* ^2^ = 8.48 with 16 degrees of freedom; *p* = 0.933). Therefore, the grades of the blastocysts transferred on day 5 were not associated with the hCG groups. Hence, blastocyst grades were not associated with serum hCG levels determined the morning following the hCG injection (Figure 1(c)).

### 3.4. Clinical Pregnancy

**Figure 2:**
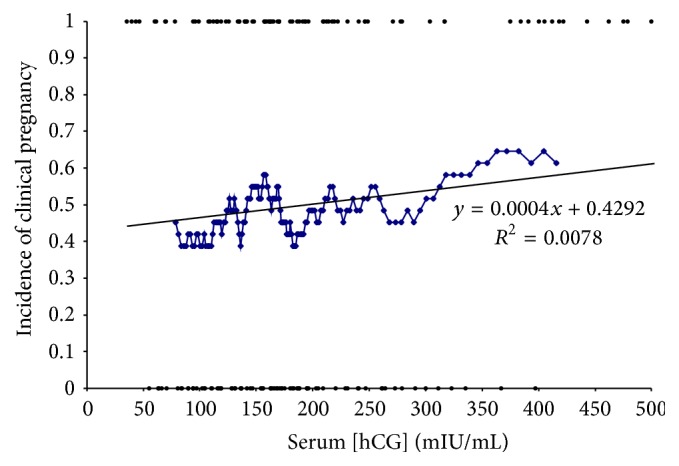
Postovulatory serum hCG level and pregnancy rate. The incidence of pregnancy was 50%. Serum hCG levels the morning after ovulatory injection did not correlate with incidence of clinical pregnancy (*R* = 0.088). Patients who were pregnant were considered a value of 1 and patients who were not pregnant were considered a value of 0. Straight line is the regression line for all patients. The blue tracing displays a rolling average of the patients.

Two hundred five of the retrievals resulted in clinical pregnancy (205/472 = 43.4%). Clinical pregnancy was not significantly correlated with hCG levels determined the morning following the hCG injection (*R* = 0.088, *p* = 0.28) (Figure 2). In addition, there is no apparent threshold for postovulatory serum hCG levels that will predict pregnancy. The four lowest serum hCG values (35.6, 37.5, 43.7, and 46.6 mIU/mL) were all associated with a clinical pregnancy (4 adjacent points, upper left in Figure 2).

### 3.5. Time of Implantation

**Figure 3:**
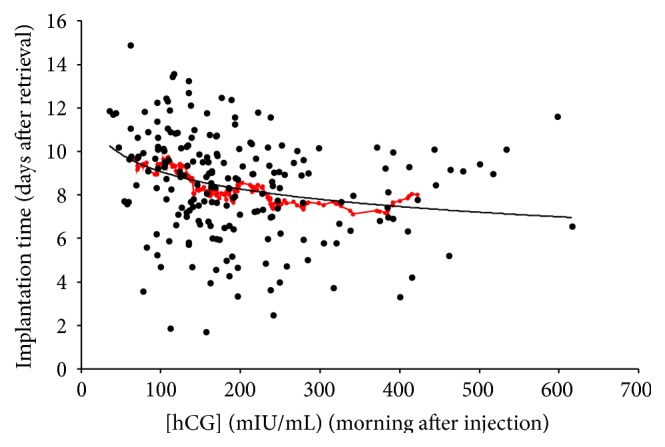
Time to implantation (days) as a function of serum hCG level the morning after 10 000 units ovulatory injection. The mean time to implantation was 8.6 ± 2.3 days (*n* = 113). There was an inverse correlation between implantation time and serum hCG (*R* = −0.335). A rolling average of the implantation time (red circles) was longer (9–10.5 days) when the postinjection serum hCG levels were lower (~100 mIU/mL) when compared to an implantation time of ~8 days when the postinjection serum hCG levels were higher (250–400 mIU/mL). There was a significant inverse association (regression equation, smooth line) between implantation time and serum hCG level (*p* = 9 × 10^−7^).

The mean time to implantation was 8.6 ± 2.3 days. Implantation times ranged from 3.2 to 14.9 days after oocyte retrieval. Roughly 72% of the implantations occurred on day 6, 7, 8, 9, or 10. The mean time to implantation was later (9.5–10 days) when the postinjection serum hCG level was low (~100 mIU/mL) when compared to 8 days when the postinjection serum hCG level was higher (250–400 mIU/mL). Despite the wide degree of variability of implantation times, the trend toward earlier implantation time with higher serum levels of hCG seems apparent even in the raw data points. A semilogarithmic best-fit line fits the raw data well and mirrors the trend present in the rolling average. This inverse relationship between logarithm of time to implantation and serum hCG level yielded a significant correlation coefficient (*R* = −0.335; *p* < 0.001) (Figure 3).

### 3.6. Association between Time of Implantation and Postinjection Serum hCG Levels and Patient Weight

HCG levels varied from patient to patient. One possible contributor to the variation in hCG levels was the final volume of dilution within the patient, a value proportional to 1/patient weight. Serum hCG levels were significantly correlated with 1/patient weight (*r* = 0.62, *p* < 0.001). In addition, the time of implantation was significantly correlated with 1/patient weight (*r* = 0.36, *p* < 0.001). Therefore, 1/patient weight, postinjection hCG levels, and the time of implantation were all significantly associated.

In order to attempt to discern if dilution factor could explain both the postinjection hCG levels and the estimate of implantation time (based on serum levels of hCG), we corrected the implantation time estimates by using individualized thresholds of serum hCG for each patient. These individualized thresholds were determined by multiplying the original threshold (10 mIU/mL per fetal sac) by the dilution factor, 156.27 lb/patient weight (a dimensionless factor obtained by dividing the average weight of all patients (156.27 lb) by the specific patient's weight). When this correction was applied, the association between postinjection hCG level and corrected implantation time was not significant (*r* = −0.07, *p* = 0.31)

## 4. Discussion

To our knowledge, this is the second published report examining postinjection serum *β*-hCG levels and IVF outcome. It has been previously reported that improper administration of the ovulatory hCG results in a serum hCG level of zero and the empty follicle syndrome where no oocytes are obtained at retrieval [2–5]. One might expect that different serum levels of hCG could lead to different rates of ovulation, different attainment of maturation by oocytes, and possibly different rates of embryonic development.

The time of implantation was significantly associated with serum hCG level the morning following hCG injection. This novel observation is particularly revealing in consideration of the observation that there were no significant differences in embryonic development. The actual time to implantation cannot be determined accurately without the use of histological examination, impossible to perform during a cycle of conception. Hence, hCG level evaluation (serum and/or urine) has traditionally been used as secondary variable to evaluate time of implantation [6–10].

Our observations reveal an expected relationship between weight and dilution of administered drug. Causal roles of weight versus hCG level on implantation time are difficult to establish when multiple variables are associated with the outcome as well as with each other. One prior study (Shah et al., [11]) found no significant association between obesity status and hCG levels following intramuscular injection examining smaller numbers of subjects. While it is plausible that weight was a major determinant of serum hCG level, and that postinjection hCG levels were determinants of the time of implantation, it is not possible to establish from these data whether implantation time was affected by the hCG level attained following hCG injection, or by patient weight or some other unidentified factor that may be associated with one, two, or all three of these parameters. This work suggests that patient weight may be a confounding factor in the use of this technique to estimate the time of implantation. The observation that correction of the implantation time by adjusting the hCG threshold for the dilution factor suggests that the same dilution effect that occurs for exogenous injection of hCG may occur with the endogenous release of hCG. When adjustment for this was applied, the association of implantation time with postinjection hCG level was not significant, suggesting that the serum hCG levels shortly following implantation are diluted to the same extent as injected hCG. Further, this suggests that the hCG levels expected during pregnancy should be adjusted by patient weight. Dismissal of a pregnancy as unsuccessful based on a single low serum hCG level in a heavy patient may lead to poor patient management.

Despite the initial *β*-hCG and appropriate doubling in forty-eight hours, the exact time of implantation is still quite unpredictable. Previously, several studies have been conducted to shed light on this phenomenon. Wilcox et al. collected daily urinary hCG samples for six months in 221 women attempting natural conception [7]. In 199 natural conceptions, they found that implantation day (the first day that hCG appeared in the woman's urine) ranged from day 6 to day 18 after ovulation and that 84% of the women had implantation on day 8, 9, or 10 following ovulation [7]. The risk of early pregnancy loss increased with later implantations [7]. The range of implantation times that we report after oocyte retrieval (3.2 to 14.9 days after oocyte retrieval) is quite similar to the range of implantation days after ovulation reported by Wilcox et al. [7]. Our observation that 72% of the implantations occurred on day 6, 7, 8, 9, or 10 also suggests that implantation following IVF occurs with a similar interval after hCG injection as implantation occurs following the LH surge in the natural cycles observed by Wilcox et al. [7].

In laboratory animals it has been demonstrated that the endometrium goes through several phases: (1) neutral toward implantation, (2) receptive window of implantation, and (3) refractory phase to implantation [12, 13]. It is still unclear exactly what histological and morphological endometrial change as well as what hormonal milieu is optimal for implantation. Generally, based on donor and frozen embryo transfer cycles, it is clear that some level of endometrial receptivity (endometrial histologic readiness in the right hormonal milieu) is necessary for implantation to occur. Based on our findings here, embryo developmental extent and quality were not associated with serum hCG levels the morning following the hCG injection. However, time to implantation was inversely related to these serum hCG values. This may suggest that that the hCG levels attained following the ovulatory injection of hCG affect the endometrial window of receptivity (and hence time to implantation).

We did not evaluate if postinjection serum hCG values or if time to implantation was affected by the type of stimulation protocol. However, it has been previously reported that time to implantation is not affected by the type of IVF stimulation protocol used. It was previously demonstrated that implantation time did not differ among women using protocols of (a) IVF stimulation without a GnRH agonist, (b) long GnRH agonist protocol and embryo transfer on day 2, (c) long GnRH agonist protocol and embryo transfer on day 3, and (d) GnRH flare protocol [6].

In summary, we found that postinjection serum hCG levels did* not* correlate with number of oocytes retrieved per follicle scanned, maturity of the retrieved oocytes, embryo development or quality, or incidence of pregnancy confirming the results of Levy et al. [14].

Postinjection serum hCG levels determined were inversely correlated with both the time of implantation and patient weight. Higher serum hCG levels were associated with earlier implantation times. However, the lack of significant correlation when implantation time was corrected using individualized hCG thresholds adjusted for patient weight suggests that the association between implantation time and postinjection serum hCG levels may be due to similar dilution of exogenous and endogenous hCG levels. Since the extent of embryonic development was not different at different serum hCG levels, we propose that embryonic developmental rate or quality observed at the time of embryo transfer was not responsible for these differences in the time of implantation. We propose a larger series to confirm these observations, along with further investigations to better determine the exact mechanism(s) of this phenomenon, before this information might be used in attempts to improve clinical outcomes.

## Capsule

Postinjection serum hCG level predicts neither embryo quality nor IVF outcome. However, it is closely associated with implantation time, and with patient weight. After correction for patient weight in calculations for implantation time, the association of implantation time with the postinjection hCG level is no longer significant, suggesting that serum hCG levels are dependent upon patient weight both following hCG injection and during endogenous rises of hCG during early pregnancy.
